# Effect of 90Sr internal emitter on gene expression in mouse blood

**DOI:** 10.1186/s12864-015-1774-z

**Published:** 2015-08-07

**Authors:** Shanaz A. Ghandhi, Waylon Weber, Dunstana Melo, Melanie Doyle-Eisele, Mashkura Chowdhury, Raymond Guilmette, Sally A. Amundson

**Affiliations:** Center for Radiological Research, Columbia University Medical Center, VC11-215, 630 West 168th Street, New York, NY 10032 USA; Lovelace Respiratory Research Institute, Albuquerque, NM 87108 USA

**Keywords:** Gene expression, Microarrays, Strontium-90, MicroRNA, Radiation biodosimetry

## Abstract

**Background:**

The radioactive isotope Strontium-90 (^90^Sr) may be released as a component of fallout from nuclear accidents, or in the event of a radiological incident such as detonation of an improvised nuclear device, and if ingested poses a significant health risk to exposed individuals. In order to better understand the response to ^90^Sr, using an easily attainable and standard biodosimetry sample fluid, we analyzed the global transcriptomic response of blood cells in an *in vivo* model system.

**Results:**

We injected C57BL/6 mice with a solution of 90SrCl2 and followed them over a 30-day period. At days 4, 7, 9, 25 and 30, we collected blood and isolated RNA for microarray analyses. These days corresponded to target doses in a range from 1–5 Gy. We investigated changes in mRNA levels using microarrays, and changes in specific microRNA (miRNA) predicted to be involved in the response using qRT-PCR. We identified 8082 differentially expressed genes in the blood of mice exposed to ^90^Sr compared with controls. Common biological functions were affected throughout the study, including apoptosis of B and T lymphocytes, and atrophy of lymphoid organs. Cellular functions such as RNA degradation and lipid metabolism were also affected during the study. The broad down regulation of genes observed in our study suggested a potential role for miRNA in gene regulation. We tested candidate miRNAs, *mmu-miR-16*, *mmu-miR-124*, *mmu-miR-125* and *mmu-mir-21*; and found that all were induced at the earliest time point, day 4.

**Conclusions:**

Our study is the first to report the transcriptomic response of blood cells to the internal emitter ^90^Sr in mouse and a possible role for microRNA in gene regulation after ^90^Sr exposure. The most dramatic effect was observed on gene expression related to B-cell development and RNA maintenance. These functions were affected by genes that were down regulated throughout the study, suggesting severely compromised antigen response, which may be a result of the deposition of the radioisotope proximal to the hematopoietic compartment in bone.

**Electronic supplementary material:**

The online version of this article (doi:10.1186/s12864-015-1774-z) contains supplementary material, which is available to authorized users.

## Background

The field of radiation biodosimetry seeks to identify the best and most practical methods for detecting exposure to radiation; and estimating dose and the potential health risk to an exposed individual. Gene expression approaches are being developed to enhance the ability to establish exposure doses [1–6] and suggest treatment modalities appropriate to the time and dose received. However most of this data has been collected, and models built, using exposure to gamma radiation. There is very little information regarding how exposures from internal emitters could affect dose estimates from large-scale radiation biodosimetry approaches. This is of concern, as in most large-scale radiological scenarios significant exposure to inhaled or ingested radionuclides would be expected [7–10]; and these could alter the medical prognosis relative to acute external exposures. Strontium-90, decays by beta emission to Yttrium. Because neither radionuclide emits photon radiation they are considered to be pure beta emitters. Strontium is a bone-seeker, as is Yttrium, and is deposited mainly in bone. Because of the beta emissions and the radionuclide deposition in bone, most of the committed dose is delivered in skeleton tissue and bone marrow. With a half-life of 29 years and the shorter penetration range of beta decay, intake of ^90^Sr would be expected to result in long-term exposure spanning the lifetime of an individual [11–13].

After the Chernobyl accident, ^90^Sr was found to contaminate food and soil and was also internally deposited in people exposed to fallout [8, 11, 14]. People living along the Techa River in Russia were also exposed to ^90^Sr as well as other radionuclides that were dumped into the river over an extended period during the Cold War. Individuals exposed to a mean average dose of 100-200 mGy/year during the years of maximum exposure (1950–56) were found to show inhibition of hematopoiesis [15], and ongoing epidemiological studies have shown excess relative risks for both leukemia [16] and solid cancers in this population [17].

Cancer incidence has also been studied in mice exposed to ^90^Sr as an internal emitter, and no tumors were observed below a cumulative average bone dose of 18 Gy [18], suggesting that structural and cellular effects on bone and hematopoietic functions may be the highest risks from internal contamination by ^90^Sr in mice [19–22]. Other mouse studies have reported ^90^Sr effects including altered gene expression and cellular changes in bone cells [20, 22, 23]. These studies explored the effects of strontium ingestion, and therefore chronic exposure, in BALB/c mice, with a maximum dose of 10 mGy at the end of a 20-week study; and it was found that these doses affected bone morphogenesis, but not hematopoiesis.

The present study was designed to investigate gene expression in peripheral blood, following exposure of C57BL/6 mice to internal radioactive strontium with a delivered dose in the range similar to that used successfully in a previous study with ^137^Cs [24]. In this latter study, we have recently shown that the internal emitter ^137^Cs, injected as a soluble salt giving total body exposure, can alter gene expression in mammalian blood, affecting different biological processes across the course of a 30-day study [24]. In the ^90^Sr study, we again used injection as the mode of delivery, in order to maintain consistent doses in replicates over the 30-day study. We have shown that ^90^Sr injection into C57BL/6 mice results in changes in transcriptomic profiles of blood cells. Thousands of genes were affected, most of which were down-regulated after exposure to the radionuclide, with microRNA possibly playing a role in the coordinate down Typical radiation response genes were also induced at several times. Our study represents a first step towards identifying the gene expression response of blood to ^90^Sr. This is of importance in the development of a blood-based gene expression signature of ^90^Sr to determine its contribution to the total dose received.

## Methods

### Animals and irradiation

All animal experiments were conducted in accordance with applicable federal and state guidelines and were approved by the Institutional Animal Care and Use Committee (IACUC) of the Lovelace Biomedical and Environmental Research Institute (LBERI) and the IACUC of Columbia University (approval number AC-AAAG4356). C57BL/6 mice (approximately 10-12 weeks old, 25-30 g) were received from Charles River Laboratories (Frederick, MD) and were quarantined for 14 days prior to group assignment by body weight stratification for randomization into the study.

Animals were administered ^90^Sr intravenously by tail vein injection with 200 ± 0.3 kBq ^85/90^SrCl_2_ solution in a volume of 0.05 mL. Strontium-85 (^85^Sr) was used as a tracer for the purpose of whole body activity measurement. Strontium-85 comprised approximately 1 % of the final formulation activity. Although the addition of Sr-85 contributed gamma rays to the emitted radiation from Sr-90/Y-90, the photons added less than 0.01 % to the absorbed radiation dose to bone and bone marrow. The vehicle saline solution was delivered to the control group. Measurements obtained from the animals and tissue samples were compared to the calibration curves of ^85/90^Sr to determine the amount of ^90^Sr present. Calibration curves were generated using known amounts of Sr isotopes, which were placed into phantoms with geometry consistent with that of the mouse. Phantoms were analyzed in the same manner as for mice and tissue samples. The limit of detection for this radio-analytical method was 40 Bq. After dose administration, mice were housed individually in micro-isolator cages with lead shielding used to minimize cross-irradiation from adjacent mice. All animals had unlimited access to Teklad Certified Global Rodent Diet 2016 (Harlan Teklad, Madison, WI) and to water except during dose administration and whole-body *in vivo* counting. No adverse effects were noted on the animals, based on clinical observation during the course of the study.

The animals were divided into five radiation dose groups, 8 animals per group. The committed radiation doses were in a range from 1 to 5 Gy. In order to receive those committed doses, the animals were sacrificed at days 4, 7, 9, 25 and 30 after ^90^Sr intravenous administration. On scheduled necropsy days, animals were euthanatized by intraperitoneal (IP) injection of Euthasol (>150 mg/kg [390 mg/mL pentobarbital and 50 mg/mL phenytoin in sterile saline]) and weighed. Whole blood was collected by cardiac puncture in a sterile hood; a full necropsy was conducted and liver, spleen, kidneys, lungs, muscles (right and left quadriceps), GI tract (upper and lower), gonads/reproductive tract, femurs, and any soft tissue remains were collected. The brain and eyes were removed from the skull and combined with the soft tissue remains.

### Biokinetics and dosimetry of ^90^Sr in mice

Animals were radioassayed for ^90^Sr whole-body content using the LBERI *in vivo* photon counting system, consisting of dual 5″ diameter Phoswich (dual NaI (Tl) – CsI (Tl) detector and associated pulse height analysis electronics). This *in vivo* detection system was adapted from a scintillation detector system originally designed for measuring low-energy photons *in vivo* in human subjects and experimental animals [25]. Animals were placed in small containers, with breathing holes, and measured to determine the amount of radioactivity present in each animal daily on days 0-7, then on days 9, 11, 15, 16, 20, 23, 25, 27, and 30 after ^90^Sr administration (until the time of necropsy). The measurement system was calibrated for different geometries; phantoms representing the animal body and samples were developed using a ^85/90^Sr NIST-traceable standard solution. Calibration was performed each day prior to the measurement. The animals and samples were measured for 3 min. The ^90^Sr whole body retention profile was derived from whole body ^90^Sr measurements. The whole-body retention data from each mouse were fitted individually to negative exponential functions. The average whole body retention equation was determined to be:

1 $$ {\mathrm{R}}_{\left(\mathrm{t}\right)}=52.1{\mathrm{e}}^{-2.0\mathrm{t}}+20.7{\mathrm{e}}^{-0.13\mathrm{t}}+27.2{\mathrm{e}}^{-0.005\mathrm{t}} $$

Where R_(t)_ represents the whole-body content at time (t), expressed as percentage of the injected ^90^Sr activity; and t is in days. The respective biological half times were 0.3 days, 5.3 days, and 139 days.

In order to calculate the committed absorbed dose to skeleton, the dose coefficient (Gy.Bq^−1^ of administered activity) was derived using Eq. 2. The comparison between the whole-body activity and the ^90^Sr content in skeleton at sacrifice time shows that about 95 % of the whole-body activity was located in skeleton for all time periods. So the retention parameters of Eq. 1 were used to calculate the total number of nuclear transformations (Bq s) in skeleton for each time period of the study. The S value (Gy/Bq s) used in Eq. 2 was derived specifically for young adult mice and rats by Stabin et al. [26].

The committed absorbed doses to the skeleton for each animal was calculated by multiplying the dose coefficient (Gy Bq^−1^) related to the specific sacrifice time for each animal in the study by the administered activity (Bq).

2 $$ \frac{D_T}{A}={\displaystyle {\int}_{t_0}^{t_{0+t}}\overset{\smile }{\mathrm{A}}}(s)\times S\left({r}_T\leftarrow {r}_S,t\right)\left(\frac{Gy}{Bq}\right) $$

Where *Ã*(*S*) is the time-integrated activity (Bq s), equal to the total number of nuclear transformations in the source region (skeleton); S (r_T_ ← r_S_, t), in Gy per Bq s, is the S value from r_S_ to r_T_ of ^90^Sr + ^90^Y, where the S value for a given source (r_S_) -target (r_T_) pair is the mean absorbed dose to the target organ per ^90^Sr + ^90^Y total number of nuclear transformations in the source region.

The current study delivered whole body absorbed doses to the animals of approximately 1.2, 1.8, 2.1, 4.8 and 5.3 Gy. All animals were injected with the same activity of ^90^Sr, and the euthanasia time points post ^90^Sr administration were selected to result in the desired range of whole-body doses to the mice.

Since most soft tissues contained Sr-90 concentrations that were < 1 % those measured in bone, it was assumed that all material in the whole body was found in bone. This was essentially the case after 1 day. In addition, since all animals were consistent in their whole-body counts, and there were no outliers, all dose calculations were conducted from an initial whole body burden and using the same average elimination curve. Animals in the day 4 group received an average dose of 1.2 ± 0.1 Gy, the day 7 group received an average dose of 1.8 ± 0.1 Gy, the day 9 group received an average dose of 2.1 ± 0.3 Gy, the day 25 group received an average dose of 4.8 ± 0.4 Gy, and the day 30 group received an average dose of 5.3 ± 0.7 Gy (Table 1).

**Table 1:** Summary of gene expression changes across the study

Time (day)	Dose (Gy)	Dose rate (Gy/day)	No. genes differentially expressed	No. genes down-regulated (%)	Percentage genes overlap with following time
4	1.2	0.30	3957	3580 (91 %)	56
7	1.8	0.20	3023	2633 (87 %)	68
9	2.1	0.15	3584	3122 (87 %)	56
25	4.8	0.17	3478	2683 (77 %)	74
30	5.3	0.10	5768	4431 (77 %)	NA

### Microarrays and analysis

All blood samples were collected by cardiac puncture in a sterile hood. For each animal ~0.4 mL of blood was collected without anti-coagulant and placed directly into 2 mL of PAXgene Blood RNA stabilization and lysis solution (PreAnalytix GmBH, catalog#762165), mixed thoroughly and shipped at 4 °C in temperature-stabilized containers. Blood smears were made at the time of blood collection and manually counted, there were no significant changes between blood cell counts of control and irradiated mice at any time (*p*-value >0.05 for all pairs; Additional file 1). The blood samples in PAXgene solution were stored for a further 24 h at 4 °C, and then incubated at room temperature for a minimum of 2 h before proceeding with RNA isolation. RNA was purified following the PAXgene Blood RNA kit recommended protocol with on-column DNaseI treatment. Globin transcripts were reduced using the Ambion GLOBINclear-Mouse/Rat kit (Life Technologies, Grand Island, NY, catalog# AM1981). RNA yields were quantified using the NanoDrop ND1000 Spectrophotometer (Thermo Scientific) and RNA quality was checked using the 2100 Bioanalyzer (Agilent Technologies). RNA used for microarray hybridization had an average RNA Integrity Number of 8.2.

Cyanine-3 (Cy3) labeled cRNA was prepared with the One-Color Low Input Quick Amp Labeling Kit (Agilent Technologies) according to the manufacturer’s instructions. Dye incorporation and cRNA yield were checked with the NanoDrop ND1000 Spectrophotometer; 1.6 μg of cRNA, >9 pmol Cy3 per μg cRNA was fragmented and hybridized (17 h with rotation at 65 °C) to Agilent Whole Mouse Genome Microarrays 4X44K v2 (G4846A), and then washed using the Gene Expression Hybridization Kit and GE Wash Buffers as recommended by Agilent. Slides were then scanned with the Agilent DNA Microarray Scanner (G2505B), and the images were analyzed (Agilent Feature Extraction Software ver. 10.7) with default parameters for background correction and flagging non-uniform features.

Background-corrected hybridization intensities were imported into BRB-ArrayTools, Version 4.2.1 [27] log2-transformed and median normalized. Non-uniform outliers or features not significantly above background intensity in 25 % or more of the hybridizations were filtered out. A further filter requiring a minimum 1.5 fold change in at least 20 % of the hybridizations was then applied, probes were averaged to one probe per gene and duplicate genes were reduced by selecting the one with maximum signal intensity, yielding a final set of 14,208 features that were used for subsequent analyses. The microarray data is available through the Gene Expression Omnibus with accession number GSE64775.

RNA from six individual mice for each time point and condition (with the exception of day 4 samples, for which only four replicates were available) were hybridized. Class comparisons were conducted using BRB-Array Tools to identify genes that were differentially expressed between controls and ^90^Sr-exposed animals at each of the five sacrifice times using a random-variance *t*-test [28]. Genes with p-values less than 0.001 and an additional fold change cut off of ±2 were considered statistically significant. The false discovery rate (FDR) was estimated for each gene using the method of Benjamini and Hochberg [29], to control for false positives. All genes used in this analysis had a false discovery rate of <0.002.

### Quantitative PCR of mRNA and microRNA

The High-Capacity cDNA Archive Kit (Life Technologies, Foster City, CA) was used to prepare cDNA from total RNA. Quantitative real-time RT-PCR (qRT-PCR) was performed for selected genes using Taqman assays (Life Technologies) to confirm microarray experiment findings for the selected genes. Assays for genes were as follows: *Mt2* (assay ID: Mm04207591_g1); *Unc93b1* (assay ID: Mm00457643_m1); *Cdkn1a* (assay ID: Mm01303209_m1); *Ccng1* (assay ID: Mm00438084_m1); *Ddb2* (assay ID: Mm1333909_m1) and *Bbc3* (assay ID: Mm00519268_m1). In gene expression validation studies, 20 ng cDNA was used as input for replicate reactions. Quantitative real time PCR reactions were performed with the ABI 7900 Real Time PCR System using Universal PCR Master Mix (Life Technologies), with initial activation at 50 °C for 120 s and 95 °C for 10 min, followed by 40 cycles of 95 °C for 15 s and 60 °C for 60 s. Relative fold-induction was calculated by the ΔΔC_T_ method, using SDS version 2.3 (Thermofisher). Data were normalized to *Actb* gene expression levels, which were found to be stably expressed across all samples.

Specific miRNA were also measured using Taqman miRNA assays (Life Technologies). RNA of four miRNA species were measured: *mmu-miR-16* (assay ID: 000391); *mmu-miR-124* (assay ID: 001182); *mmu-miR-125* (assay ID: 000449) and *mmu-miR-210* (assay ID: 000512), following the recommended protocol from Life Technologies. Relative fold-induction was calculated by the ΔΔC_T_ method, using SDS version 2.3 (Thermofisher). Data were normalized to *U6snRNA* (Life Technologies, catalog number 4427975) expression levels.

All qRT-PCR results are shown as average of biological replicate samples using unpaired analyses and p-values were computed (using two-sample *t*-test assuming unequal variances) for differences between irradiated and control samples at every time point measured. All fold changes >2 and <0.5 were significant (*p*-value <0.05) with the exception of *Cdkn1a* at day 4 (*p*-value > 0.05).

### Gene ontology and pathway analysis

Lists of genes significantly over- or under-expressed relative to controls were imported separately into the Database for Annotation, Visualization and Integrated Discovery (DAVID) ver 6.7 to identify enriched biological themes and gene ontology (GO) terms using the functional annotation tool [30]. Benjamini corrected *p* values <0.05 were considered significant.

The significantly differentially expressed gene lists from each sacrifice time, along with their relative expression levels, were also imported into Ingenuity Pathways Analysis (IPA) (Ingenuity® Systems, http://www.ingenuity.com) and analyzed with the IPA Core Analysis Tool. IPA uses curated information on the published relationships between gene products to predict networks and associations between genes in a list. The upstream regulator analysis specifically uses information about the relationship between the activity of potential upstream regulatory factors (transcription factors, cytokines, receptor mediated nuclear factors and microRNA were chosen for these analyses) and the expression changes of the measured genes to make predictions on the regulatory status of the upstream molecule. IPA generates a z-score for each factor in the upstream regulator analysis and for prediction of activation or inhibition state of biological functions. The IPA default cutoff of z > 2 was used to predict activation and z < −2 to predict inhibition.

## Results

**Fig. 1:**
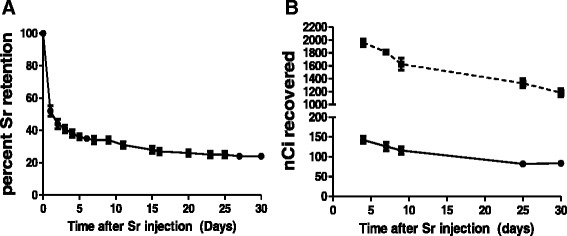
Analysis of ^90^Sr retention for dose calculation. **a** Retention of ^90^Sr over the time course of the study, points are whole-body counts of ^90^Sr activity shown as percentage of the injected amount; measured daily on days 0-7, then on days 9, 11, 15, 16, 20, 23, 25, 27, and 30 after injection. Points are average counts and error bars are SEM of animals measured over the time course. **b** Amount of ^90^Sr activity recovered after necropsy in femur (right and left) and carcass (not de-boned containing the remaining skeletal tissue). The average content on day 4 was 142 nCi, which decreased to 126 nCi on day 7 and 116 nCi on day 9. Femur content (●) stabilized for the last two collection times. Carcass content (■) on the day of euthanasia and necropsy was consistent with collected whole-body counts and decreased for the duration of the study. All points are average and SEM measurements of ^90^Sr activity measured in necropsied animals on days indicated

Animals in this study were weighed before necropsy on days 4, 7, 9, 25 and 30 and no significant differences were observed in weight between control and ^90^Sr-injected groups. Dose rates in this study were relatively low, ranging from an average of 0.3 Gy/day from injection to the earliest time of necropsy, day 4, to an average of 0.1 Gy/day between days 25 and 30, representing a change by a factor of 3 across the study time course (Table 1). The average accrued dose at the 30-day time point was 5.3 Gy (Table 1). The relative retention of ^90^Sr over the course of the experiment is shown in Fig. 1a and reflects the rapid loss of activity that occurred in the first 24 h after injection, when the amount of ^90^Sr fell to 52.0 ± 3.3 % of the initial amount, followed by a more gradual loss of ^90^Sr through the rest of the study and hence more stable dose rates. At necropsy, after blood samples were collected, individual organs and carcass were measured for ^90^Sr activity and the skeleton (represented by the right and left femur) and carcass (containing the rest of the skeleton) contained most of the radioactive strontium (Fig. 1b).

### Microarray results

**Fig. 2:**
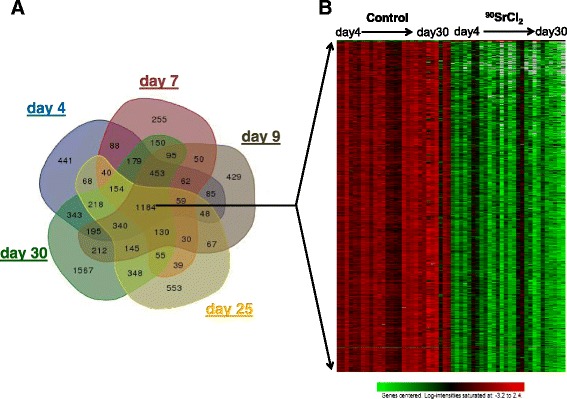
Summary of microarray gene expression results in blood cells after ^90^Sr injection. **a** Venn diagram of differentially expressed genes at each time point showing numbers of genes overlapping between time points (the Venn diagram was generated using the online tool at bioinformatics.psb.ugent.be/webtools/Venn/). There were 1184 genes that were common to all time points. **b** Heatmap of expression of the 1184 genes common across the study revealed that the majority of genes (1180 out of 1184) were down regulated rapidly after ^90^Sr injection, and stayed repressed throughout the study

At each time point, days 4, 7, 9, 25 and 30, blood was collected from mice and RNA was isolated. RNA was processed to cRNA labeled with Cy3 and hybridized to whole genome mouse arrays using the Agilent platform and recommended protocols. We used BRB-ArrayTools [27] to collate the project; and after filtering the data 14,208 genes remained that were used for further analysis steps. Class comparisons were made between control and irradiated mouse blood samples at each time point, and the resulting gene expression changes and trends are summarized in Table 1. A large number of genes were changed, with a total of 8082 genes over the 30-day time course (Additional file 2). The number of differentially expressed genes at the individual time points and the relative overlap with genes at subsequent times in the study indicated that although there were genes that were affected similarly across the time course, a large proportion of genes were specific to a time/dose, suggesting that unique biological functions were being activated or repressed at different times in the study. We also found that 1184 genes were significantly differentially expressed at all 5 time points (Fig. 2a). The majority of these genes, (1180 out of 1184) were down regulated throughout the study (Fig. 2b), with corresponding levels in control mice remaining stable over the 30 days. See Additional file 3 for a list of these genes and a brief summary of the PANTHER biological processes analysis.

Gene ontology analysis of the 1184 genes common to all times revealed top pathways affected at the gene expression level. These were KEGG pathways, mmu04662: B-cell receptor signaling pathway (19 genes changed, Bonferroni p-value 6.8 ×10^−5^), mmu04672: intestinal immune network for IgA production (13 genes changed, Bonferroni p-value 7.0 ×10^−3^); and mmu03018: RNA degradation (13 genes changed, Bonferroni *p*-value 2.1 ×10^−2^) and mmu05340: Primary immunodeficiency (10 genes changed, Bonferroni *p*-value 2.4 ×10^−2^).

**Fig. 3:**
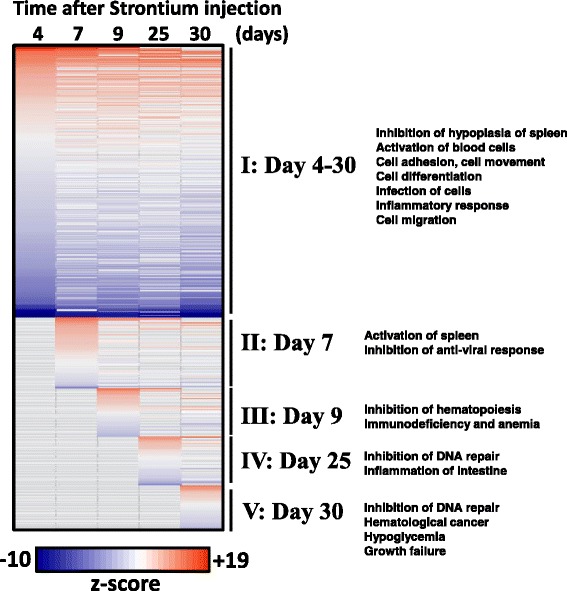
IPA based enrichment of biological functions; the heatmap shows the top scoring biological functions across the study. Each column indicates z-scores corresponding to enriched biological terms for the set of differentially expressed genes at the time indicated after ^90^Sr injection. Columns were sorted by significant processes from early (day 4, group I) to late (day 30, group V), in the study. Group I included processes that were affected across the study. Group II processes were affected starting on day 7; Group III processes, on day 9; Group IV, started at day 25, and Group V were significant only at day 30 in the study. The colors of the cells indicate z-scores, potentially activated/promoted processes (varying shades of orange) and potentially inhibited/suppressed processes (varying shades of blue); in each case increasing darkness of color represents increasing significance. Processes that did not appear in the core analysis result are shown in gray. The complete table with scores is in Additional file 4

We then analyzed all genes affected at the 5 time points of this study using the IPA biological functions tool to assess and compare categories of biological processes activated or inhibited in the course of the study, as predicted from gene expression changes. These are shown in Fig. 3. The heat map shows the scale of predicted activation (shades of orange) and inhibition (shades of blue) states for each biological function across the 5 times measured in this study. There were many processes predicted to be significantly affected across all times in the study, with most showing similar activation or inhibition across the study. These processes are sorted by decreasing z-scores and time point, shown in Fig. 3. There were many processes that were significant starting at day 4 and then mostly significant at other time points (group I); in which the top activated processes were atrophy of lymphatic systems, apoptosis of B and T cells, activation of lysosomal storage and autoimmune disease. Top inhibited processes within this group affected cell survival, cell viability, infection and migration of cells. More specifically, the gene expression changes belonged to potentially inhibited functions centered on apoptosis of B-cells, cell death of leukocytes and atrophy of lymph glands. Within group II, processes emerging as significant on day 7 included activation of hyperplasia of spleen and inhibition of antiviral response. Starting on day 9, (group III) biological processes affected included activation of anemia, inflammation of the body, and inhibition of B cell activation and lipid metabolism. Towards the end of the study at day 25 (group IV), new biological processes affected included activation of protein translation and inhibition of differentiation of lymphocytes. At day 30 (group V) biological processes involving activation of lymphoid cancer and inhibition of NK cell development were included. Further details are described in the legend for Fig. 3 and listed in the table in Additional file 4.

### Quantitative PCR validation of selected genes

**Fig. 4:**
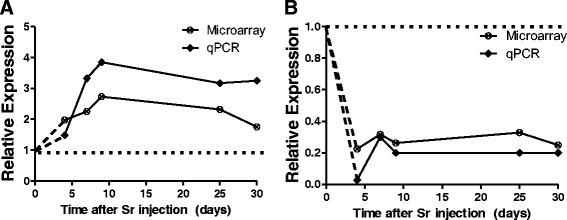
qPCR validation of microarray results for *Mt2* **a** and *Unc93b1* **b**, these genes were selected as representatives of consistently up (*Mt2*) and down (*Unc93b1*) regulated genes in the study. Relative gene expression measurements by qRT-PCR showed the same pattern of expression as the microarray results. Points are average of 6 samples per condition

We validated the relative expression of two genes selected on the basis of expression changes and consistent patterns of change across the study. One gene, metallothionein 2 (*Mt2*), was up regulated across the study and the other, unc-93 homolog B1 (*Unc93b1*), was down regulated. As shown in Fig. 4a and b, the microarray and qRT-PCR measurements were consistent at all times measured although qRT-PCR measurements indicated bigger changes than the microarrays. As all gene expression changes detected and used for analysis in this study were robust (false discovery rate <0.002, and fold change cut off ±2), extensive validation of gene expression was not performed.

### Pathway analysis and upstream regulation

We visualized gene expression in networks based on biological relationships using IPA Core analysis and the IPA Comparison Tool, which groups biological processes and pathways by significance and maximum connectivity between genes and regulators. We used the Upstream Regulator Analysis Tool in IPA to predict potential mechanisms and pathways and look for trends in the gene expression changes. We used the default z-score >2 or z-score < −2 as the significance cutoff for our analyses. We looked for predictions of activity of upstream regulators that were consistent at all times, and also for those that might be switched on and off at different points in the study.

**Fig. 5:**
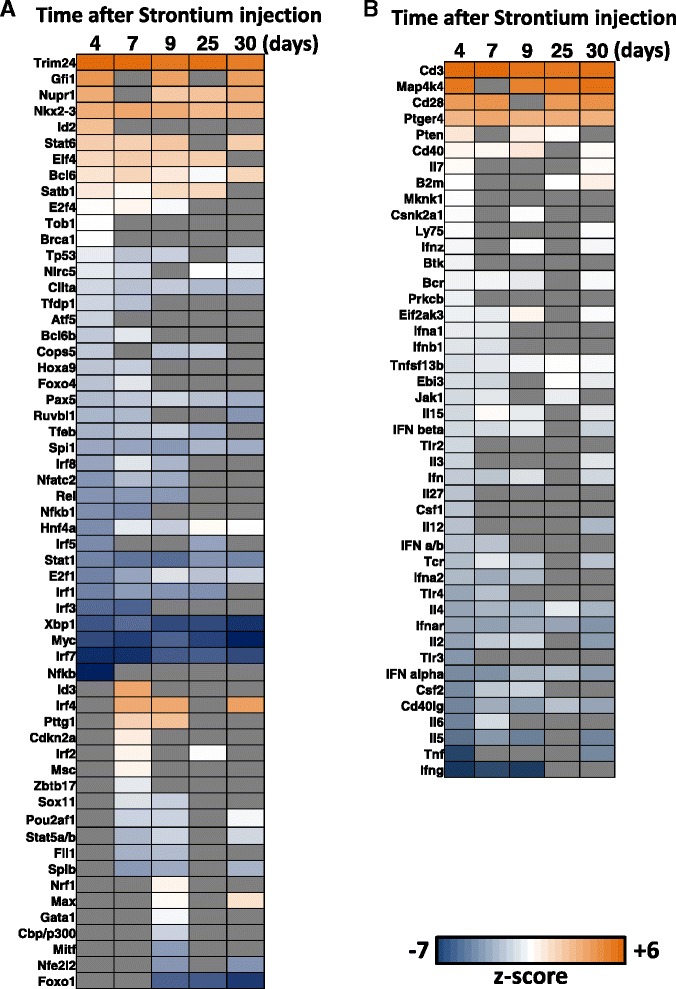
IPA-predicted upstream regulators of gene expression. **a** Heatmap of predicted activation and inhibition states of transcription regulators that may be upstream of gene expression changes seen in the study. **b** Upstream regulators categorized in IPA as cytokines, transmembrane and nuclear receptors, kinases and phosphatases. In both heat maps, colors indicate z-scores, shades of blue represent inhibition of the regulator, and shades of orange represent activation of the regulator and missing values are in gray (missing values are shown where the regulator did not have any known relationship with the significantly changed genes at that time). We used the default cut off z-score >2 or < −2 as significant for our analyses, although, z-scores between −2 and 2 are shown in the figures. The complete table with z-scores is in Additional file 5

Initially we limited the analysis to transcriptional regulators and found that a large number of transcription factors were predicted to be affected across the study. This was consistent with the large number of differentially expressed genes at each time point in our study, which suggested that multiple regulators were likely to be involved in the changing patterns of gene expression. The top candidate regulators of transcription significantly predicted to be activated at four or more times were Trim24, Nkx2-3 and Bcl6. Transcriptional regulators Gfi1, Nupr1, Stat6, Elf4, Satb1 and E2f4 were predicted to be activated at 3 or more time points in the study. The top predictions for inhibition of transcriptional regulators were Irf7, Myc, Xbp1, Irf1, Stat1, E2f1 and NFκB, all of which have been implicated in cellular stress responses. These results are summarized in Fig. 5a; the heat map shows all transcription factors that were predicted to be affected at various time points in the study, based on gene expression changes. Additionally, we also looked for the predicted status of upstream regulators categorized as cytokines, G-protein receptors, ligand-dependent nuclear receptors, transmembrane receptors, kinases and phosphatases, and found that many members of the Interferon family of proteins (Ifna1, Ifna2, Ifnb1, Ifnar and Ifnz) were predicted to be inhibited at day 4, Fig. 5b (The complete table with z-scores is in Additional file 5). Members of the Interleukin family of cytokines (Il2, Il3, Il4, Il5, Il6, Il7, Il12, Il15 and Il27) were also predicted to be inhibited between day 4 and day 9. Cd3 (Cluster of differentiation 3 and member of the T-cell receptor complex); Cd28 (Cluster of differentiation 28 and stimulator of T-cell differentiation); Mapk4 (mitogen-activated protein kinase kinase kinase kinase 4) regulators, known to be involved in TNFα signaling; and Ptger4 (prostaglandin E receptor 4 (subtype EP4), receptor for binding prostaglandin E2) were top candidates for activation in this study.

We also looked for those transcriptional regulators that may be switched on and off or vice versa at specific times in the study and found that some transcriptional regulators may be only significantly involved at one time or another and at other times silent. We found none that changed the direction of their activity during the course of the study. Interestingly, although the largest number of genes were differentially expressed at day 30 (5768 genes), the predictions suggested that there were more upstream regulators involved at other times compared with day 30. Between days 4 and 9, NFκB transcription factor dimer protein RelA was predicted to be inhibited, and in addition to other members of the NFκB family (NFkB1 inhibited at days 4 and 7) indicate that this radiation response transcription factor may be muted in blood cells.

**Fig. 6:**
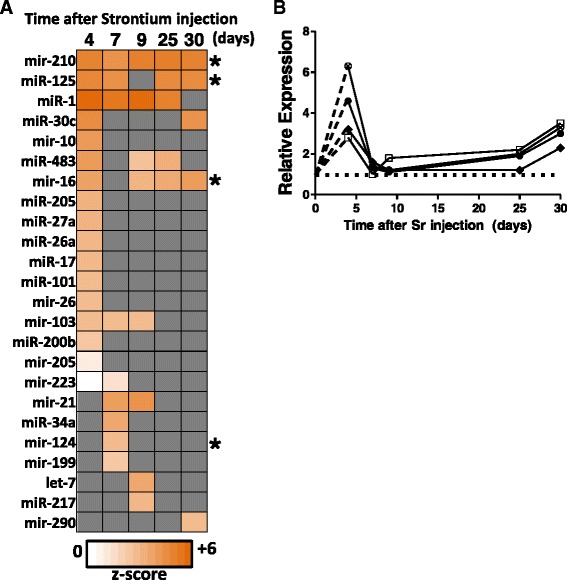
MicroRNA upstream regulators of gene expression. **a** Heatmap of predicted activation states of microRNA, which may be upstream of gene expression changes observed in the study (shades of orange, positive z-scores; missing values in gray, missing values are shown where the regulator did not have any known relationship with the significantly changed genes at that time). **b** qPCR validation of selected miRNA species: *mmu-miR-16* (⨂), *mmu-miR-124* (□), *mmu-miR-125* (♦) and *mmu-miR-210* (●). Expression peaked at the day 4 time point as predicted from the mRNA data. The complete table with z-scores is in Additional file 6. Each point is the average of 4 samples per condition

We also used IPA upstream regulator analysis to investigate potential miRNA activation. We found a number of miRNA that were predicted to be activated in the study with z-scores >2 across all time points, whereas others were only predicted to be activated at one time point, Fig. 6a. Of the 24 miRNA shown here, *mmu-miR-16*, *mmu-miR-124*, *mmu-miR-125* and *mmu-miR-210* were selected for investigation and RNA levels were measured using qRT-PCR, the result is shown in Fig. 6b (The complete table with z-scores is in Additional file 6). All microRNA tested were observed to be up regulated at day 4, with a decrease to background levels between days 7 and 9, followed by another induction peak at day 25 in the study.

## Discussion

Radioisotopes as internal emitters have been studied for their effects on human tissues and organs, used as tracers and investigated for their detrimental effects on humans after nuclear accidents. Exposure to ^90^Sr as an internal emitter is usually in the context of an accident where exposure is combined with other radionuclides, such as ^137^Cs and ^131^I [8]; and external radiation from atmosphere, soil and through contact. This makes it difficult to identify changes that may be the result of a single emitter and its possible effect on human health. Studies that have attempted to address these issues using animal models have used simple combinations of radioisotopes and one or more mode of delivery to examine the long-term effects of chronic exposure to internal emitters. In this study, ^90^Sr was injected intravenously. Using this mode of administration, we were dealing only with uncertainties related to the activity measurement as well as to the ones related to the systemic distribution and retention. We used only one injection dose, so comparison of different dose-rates at similar time points were not compared in this study.

A previously published study from our group, in which ^137^Cs was injected into mice and gene expression of blood investigated over a 30-day period, showed that a large number of genes were affected by this internal emitter [24]. The final dose in this study was 9.9 Gy at 30 days, and resulted in thousands of genes differentially expressed, with many affected biological functions found to be in common with the present ^90^Sr study. Because of Cesium’s biokinetic properties, dose rates in the ^137^Cs study were quite variable, ranging from an average of 1 Gy/day at the beginning of the study to 0.04 Gy/day between days 20 and 30. In contrast, ^90^Sr injection produced a more even low dose rate that ranged between an average of 0.3 Gy/day at the beginning of the study and 0.1 Gy/day between days 25 and 30. Many biological function terms were in common between the genes affected in blood by both ^90^Sr and ^137^Cs, however the prominent switch of genes belonging to the same biological process from up to down that was seen in the ^137^Cs study was not observed after ^90^Sr injection. The differences in gene expression response to ^90^Sr and ^137^Cs could be due to localization of the radioisotopes to different regions within the mouse body, to the different radiation quality of γ-rays and β-particles, and also to the different dose-rate gradients generated by the two isotopes after injection [24].

In another study, mice were injected with ^131^I, in which the absorbed dose was 0.1-9.7 mGy over a 24 h period, with the highest absorbed dose in the lung, which consequently had the largest number of differentially expressed genes [5]. The lowest average dose rate in the present ^90^Sr study was 100 mGy/day, a factor of 10 higher than the highest dose rate in the ^131^I study, and although different tissues were assessed for gene expression changes in the ^131^I study (lung, liver, kidney and spleen) there was an overall suppression of immune response in these animals, also seen in our results in blood.

Injection of ^90^Sr into C57BL/6 mice in our study did not have an effect on overall animal health and activity levels within the 30-day study. However, there was a dramatic effect on gene expression, starting at the earliest time measured with a relatively high number of genes changing, 3957 at day 4 (Table 1). The number of genes affected increased to 5768 genes at day 30 with at least 50 % of genes overlapping between successive time points, implying that the effect of ^90^Sr was significant throughout the study despite the lower dose rate at later times. The number of genes in common between all the time points measured was 1184, in contrast to the result from the previous ^137^Cs study, in which only 5 genes were significantly differentially expressed at all the time points measured [24]. Taken together, this suggests a strong dose-rate effect on gene expression, in addition to the effect from accrued dose. Dose-rate effects may be especially prominent in an *in vivo* model, such as mouse, where blood cells may repopulate to give rise to secondary waves of biological responses. Additionally, ^90^Sr localized to the bone, potentially affecting hematopoietic functions more strongly relative to other whole body effects as observed in the ^137^Cs study.

Functional analyses of all gene expression across the study revealed processes mostly related to immune responses, activation of apoptosis of B and T cells, changes in growth of spleen, and defects in response to infection (Fig. 3). These were the main biological changes, supported by enrichment of genes belonging to specific immune pathways that were in the 1184 genes common to all time points. Pathway terms appearing in gene ontology analysis of the 1184 common genes, comprised of the B-cell receptor signaling pathway, which results in the expression of genes that further activate the expression of other genes involved in B cell proliferation, differentiation and Ig production [31]. The second pathway involving IgA production is part of the first line of defense against microbial infection. IgA is a non-inflammatory antibody produced by an IgA plasma cell [32] and the overall repression of this pathway could indicate a reduction in the ability of the animal to neutralize toxins and pathogenic microbes. The third pathway listed includes genes involved in RNA degradation pathways that monitor the quality and turnover of RNA in cells, in order for correct protein translation to occur [33]. These processes may be compromised in blood cells after exposure to ^90^Sr, leading to further defects in biological functions involving many proteins. The fourth pathway involved primary immunodeficiency that affects cellular and humoral immunity and may be perturbed due to defective cell maturation and function during hematopoiesis [34], which could be compromised after ^90^Sr injection and deposition of the radioisotope to bone. RNA degradation pathways, also indicated to be significantly affected across the study may potentially have a widespread effect on integrity of messenger RNA of blood cells, and defective monitoring and removal of missense mRNA may result in the production of dysfunctional proteins in cells. The large proportions of down regulated genes in this study suggest that ^90^Sr localization in the proximity of the hematopoietic compartment may have compromised the development of certain subsets of the blood cells. Further investigations will be needed to confirm this.

The impact of ^90^Sr injection on gene expression was also analyzed using a prediction algorithm for upstream regulators in IPA (Fig. 5a and b). Initially we limited predictions to transcriptional regulators that recognize promoters of other genes or have a direct effect on known transcriptional factors. The top activated regulators predicted were Trim24, Gfi1, Nupr1 and Nkx2.3. The Trim24 (tripartite motif-containing 24) protein functions as an E3 ubiquitin ligase for p53 and may lead to the termination of the DNA damage response mediated by p53 [35, 36]. There is evidence that *Trim24-/-* mutant mice show adverse effects of calcium metabolism and deposition in arterioles [37] and Trim 24 activation as predicted in our study may be related to altered concentrations of strontium and calcium in the mouse. Gfi1 (growth factor independent 1) protein transcriptional factor is a known inhibitor of the p53-dependent DNA damage response in the progress of leukemia [38] and also has an important role in erythroid cell lineage development [39]. Activation of Gfi1 may be characteristic of activation of specific hematopoietic progenitor cells in response to ^90^Sr [40]. Both Trim24 and Gfi1 have known effects on p53 function in cells, leading to defects in growth and development. Another top predicted activated regulator of gene expression was Nkx2.3 (NK2 homeobox 3) protein, which is a homeodomain protein with an important role in the maintenance of the structure of the spleen [40]. It is required for proper growth of spleen fibroblast cells and may affect complement factor aggregation and immune function of the organ [41]. Activation of Nkx2.3 may indicate changes in levels of spleen growth and contribute to the changes in blood gene expression as a result of these changes.

The p53 transcription factor, although not meeting the criteria for inclusion in Fig. 5a, was predicted to be inhibited across the study (IPA generated z-scores for p53 effect on gene expression were −1.3, −2.4, −2.1 and −1.8; at days 4, 7, 9 and 30, respectively). In our ^137^Cs study, this central radiation response factor and many of its downstream effector genes were activated at early times, and then inhibited at later times [24], giving a precedent for inhibition of p53 by internal radiation emitters.

**Fig. 7:**
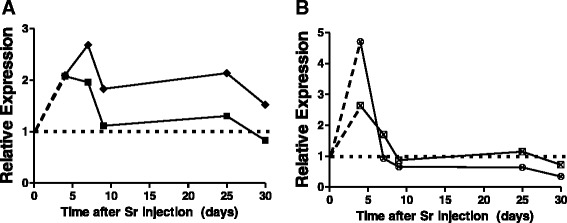
Radiation related gene expression. **a** Genes such as cyclin dependent kinase inhibitor 1 a, *Cdkn1a* (♦) and CyclinG1, *Ccng1* (■), were consistently induced across most of the time course. **b** Transcript levels for the genes Damage specific DNA binding protein, *Ddb2* (⊠) and Bcl2 binding component 3 protein, *Bbc3* (⨂), were initially induced, then back to control levels by day 9, and then stayed mostly repressed until day 30. Each point is the average of 3 samples per condition

We next looked specifically for gene expression changes of known radiation genes after ^90^Sr injection and found that mRNA for *Cdkn1a* (cyclin-dependent kinase inhibitor 1a), *Ccng1* (Cyclin G1), *Ddb2* (damage-specific DNA binding protein 2) and *Bbc3* (BCL2 binding component 3) were significantly affected at some times in the microarray data. We used quantitative RT-PCR to validate the changes in gene expression for these p53 targets and the result is shown in Fig. 7; in panel a, *Cdkn1a* and *Ccng1* transcripts were induced early and then between days 7 and 25 appeared to stabilize at a level slightly higher than controls. Panel b shows transcriptional changes in *Ddb2* and *Bbc3* genes, which were also induced at higher levels at day 4 but then repressed by day 9, after which they were repressed even further by day 30.

Upstream regulators of gene expression that were strongly predicted to be inhibited across the study were Irf7, Myc and Xbp1. Irf7 or Interferon regulatory factor 7, is a transcriptional factor produced in response to viral infections, and the regulation of Irf7 is strongly controlled because of effects on auto-immunity and cancer [41]. The predicted inhibition of Irf7 after ^90^Sr injection may indicate a reduced ability to respond to infections as reflected by changes in transcriptional targets of this protein. Xbp1, X-box binding protein 1, also predicted to be inhibited in this study, is a known regulator of MHC (major histocompatibility complex) class II molecule expression in B-cells [42]. This may have an effect on the development of B-cells *in vivo*, which is also known to occur in response to long-term ^90^Sr exposure in humans [43].

The heat map in Fig. 5b, shows the predicted states of upstream regulators of gene expression that were not categorized as transcription factors or direct repressors of transcription factors, and included G-protein receptors, ligand-mediated and transmembrane receptor proteins, kinases and phosphatase types of molecules. Of these factors, most predictions were of inhibition at day 4. An overview of the types of molecules in this list revealed that the Interferon protein family was the most prominent protein type, members of which were predicted to be inhibited at day 4 through day 9. The second most prominent group was the Interleukin protein family, also with a similar pattern of inhibition but more widespread across all times. Inhibition of Interferons may indicate defects in the hematopoietic capacity of quiescent stem cells, as studied in response to infection [44] but also possibly in response to exposure to radiation from ^90^Sr.

One of the most interesting observations in this study was that the majority of significantly differentially expressed genes at all time-points were down regulated, ranging from 91 % of all genes at day4, to 77 % of all genes at days 25 and 30 (Table 1). Additionally, there were 1180 genes common to all time points that were down regulated to similar levels across the entire study, representing an average of 33 % of the total genes affected at each time. The large number of down regulated genes and the consistent, dose-independent, changes observed at all times (Fig. 2b), suggested a possible role for microRNA-mediated repression of genes, potentially acting to suppress gene expression in a highly concerted manner [45], see also Additional file 7, which includes a network showing the microRNA and their potential target mRNA and the overlap of these networks. We selected microRNA for further testing on the basis of the strength of the prediction (z-score) and also the number of potential targets for that microRNA as predicted in IPA. We measured the transcript levels of *mmu-miR-16*, *mmu-miR-124*, *mmu-miR-125* and *mmu-miR-210* and observed the early induction of all four microRNA, consistent with their predicted state at that time, Fig. 6a and b. Between days 7 and 9, the levels of these microRNA species were seen to fall, with a slight increase again at day 25. The dynamics of microRNA induction shown here, suggest a role in early repression of target genes followed by maintenance of low target mRNA levels, however further studies are required to determine the exact role and mechanism of microRNA effects on gene regulation after ^90^Sr exposure [46, 47]. MicroRNA are known to be characteristically expressed in certain blood cell sub-sets and *mmu-miR-125* is highly expressed in T cells and neutrophils in blood [48]. MicroRNA are also known to respond to external radiation, in different cell types and also *in vivo* in blood [49–52], therefore the microRNA measured in this study may be strong candidates for further investigation for modulation of gene expression as a result of exposure to ^90^Sr. This is the first report of an effect of radiation exposure from an internal emitter on microRNA gene expression in blood.

## Conclusion

We showed that the radionuclide ^90^Sr, when injected into mice was rapidly localized to skeleton, and led to a dramatic effect on gene expression in mouse blood cells. In the course of our 30-day study, a total of 8082 genes were changed, with the majority of genes being down regulated. Pathways that were affected at all times in the study belonged to immune functions related to B-cell development and RNA maintenance, indicating that ^90^Sr had a long-lasting and widespread effect on blood cell development and also housekeeping processes in the cells. Application of prediction algorithms identified potential regulators of gene expression and suggested a possible role for microRNA-mediated gene regulation in response to this internal emitter. ^90^Sr, as an internal emitter was able to elicit a distinct response in mouse blood cells, with a large number of genes being down regulated and staying repressed throughout the study, which was very different from the response to ^137^Cs in blood. Differences in transcriptomic response may lead to identification of separate radionuclide-specific biodosimetry signatures and also impact the type of diagnosis and treatment after exposure.

### Availability of supporting data

The data set supporting the results of this article is available in the NCBI GEO repository [http://www.ncbi.nlm.nih.gov/geo/query/acc.cgi?acc=GSE64775].

## Additional files

Additional file 1:
**Manual cell counts for lymphocytes from blood smears.** (PDF 89 kb) Additional file 2:
**Differentially expressed genes in the study.** (XLS 3484 kb) Additional file 3:
**List of 1184 common genes and PANTHER GO analysis.** (XLS 143 kb) Additional file 4:
**IPA biological functions table with** **z-scores** **, details of Fig.** **3.** (XLS 99 kb) Additional file 5:
**IPA upstream biological regulators table with** **z-scores** **, details of Fig.** **5.** (XLS 33 kb) Additional file 6:
**IPA upstream regulators: miRNA, with** **z-scores**, **details of Fig.** **6.** (XLS 26 kb) Additional file 7:
**IPA networks of miRNA and potential target genes.** (PDF 699 kb)

## Abbreviations

Sr: Strontium
miR: microRNA
PCR: Polymerase chain reaction
