# Supplementary material for: Effect of 90Sr internal emitter on gene expression in mouse blood
Source: BMC Genomics. 2015 Aug 7;16(1):586. doi: 10.1186/s12864-015-1774-z (PMC4528784; doi:10.1186/s12864-015-1774-z)
Supplement: Additional file 1: — Manual cell counts for lymphocytes from blood smears. (PDF 89 kb) [file 12864_2015_1774_MOESM1_ESM.pdf]

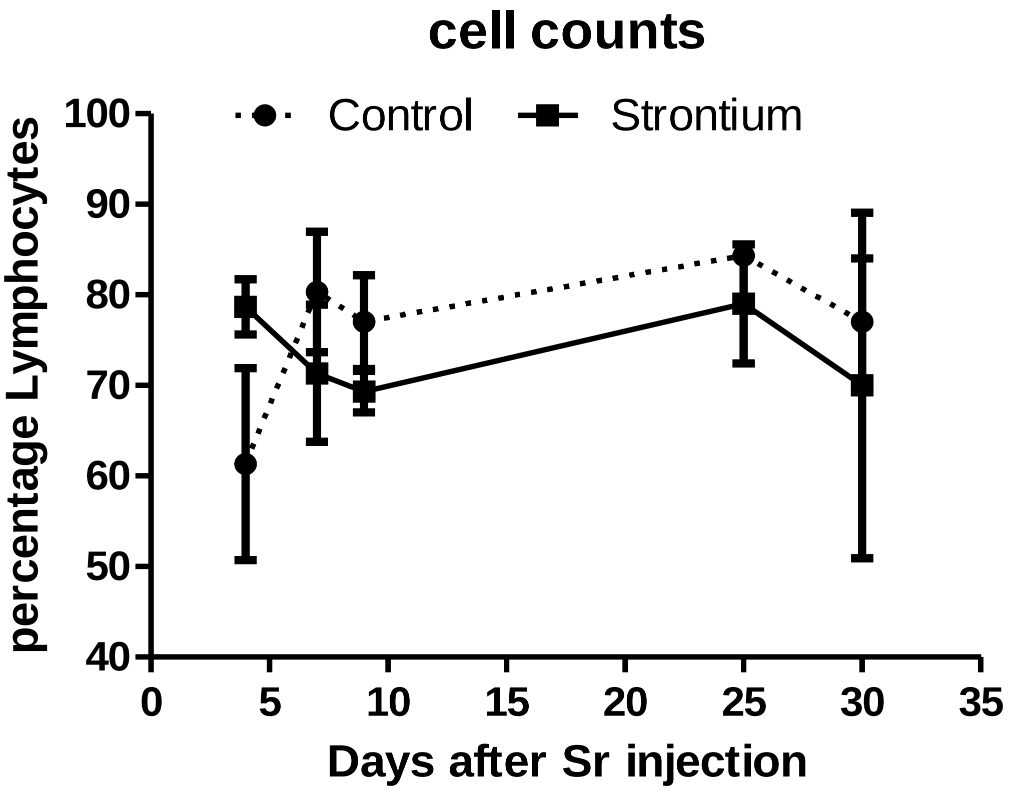

Additional file1. Blood smears were prepared at LRRI and then stained with DiffQuick and manually counted.
