# Supplementary material for: Effect of 90Sr internal emitter on gene expression in mouse blood
Source: BMC Genomics. 2015 Aug 7;16(1):586. doi: 10.1186/s12864-015-1774-z (PMC4528784; doi:10.1186/s12864-015-1774-z)
Supplement: Additional file 7: — IPA networks of miRNA and potential target genes. (PDF 699 kb) [file 12864_2015_1774_MOESM7_ESM.pdf]

Day 4

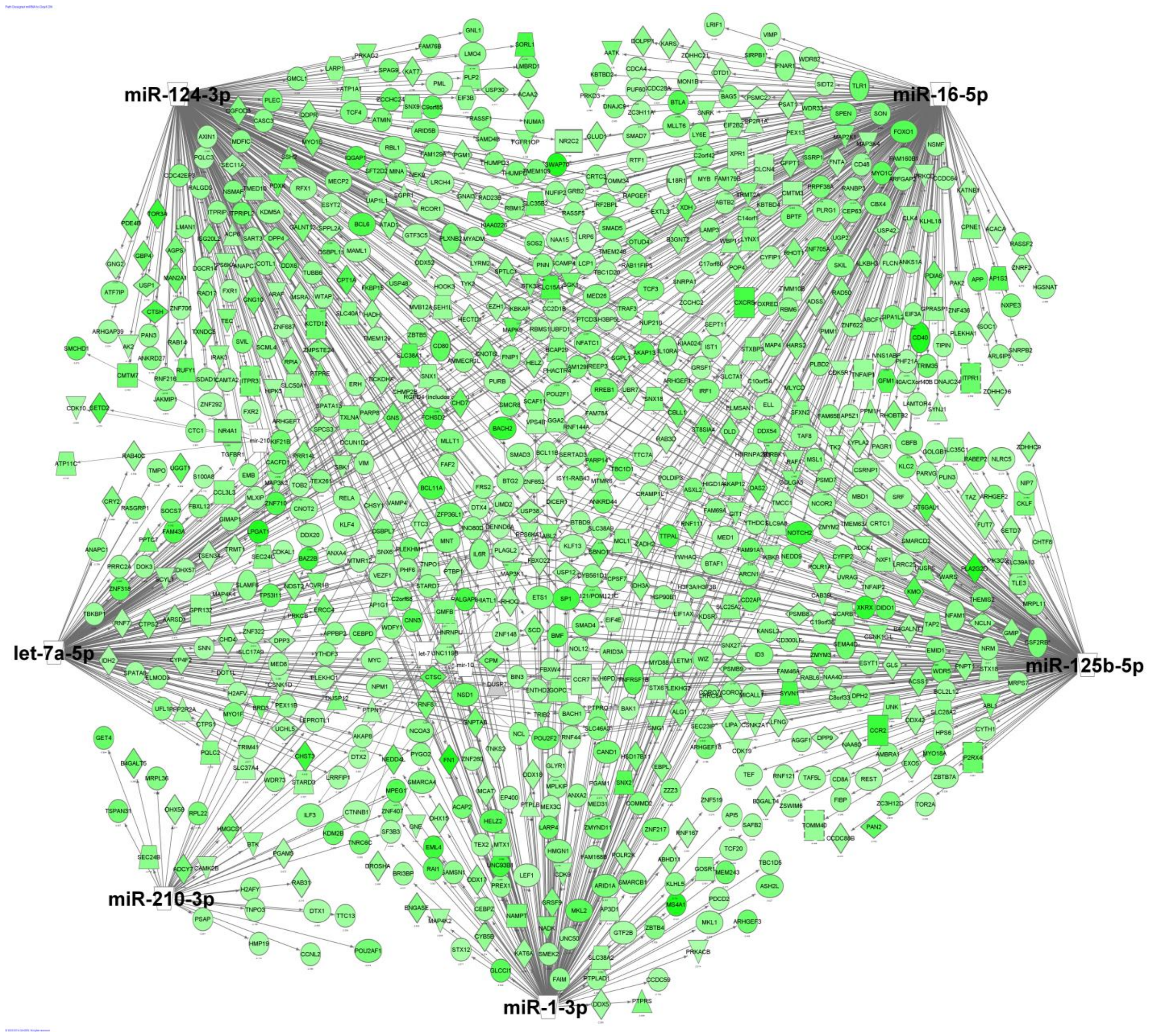

Additional file 7: Network of top predicted microRNA and potential target genes. Overlay of colors represent down-regulation of mRNA at day 4.
